# Selection of functional EPHB2 genotypes from ENU mutated grass carp treated with GCRV

**DOI:** 10.1186/s12864-021-07858-x

**Published:** 2021-07-07

**Authors:** Meher un Nissa, Zhu-Xiang Jiang, Guo-Dong Zheng, Shu-Ming Zou

**Affiliations:** 1 Genetics and Breeding Center for Blunt Snout Bream, Ministry of Agriculture, Shanghai, 201306 China; 2 Key Laboratory of Freshwater Aquatic Genetic Resources, Ministry of Agriculture, Shanghai, 201306 China; 3 National Demonstration Center for Experimental Fisheries Science Education, Shanghai Ocean University, Shanghai, 201306 China

**Keywords:** BSA, Mutant grass carp (ENU), GCRV, *q*PCR, SNP

## Abstract

**Background:**

*N*-ethyl-*N*-nitrosourea (ENU) mutagenesis is a useful method for the genetic engineering of plants, and the production of functional mutants in animal models including mice and zebrafish. Grass carp reovirus (GCRV) is a haemorrhagic disease of grass carp which has caused noteworthy losses in fingerlings over the last few years. To overcome this problem, we used ENU mutant grass carp in an attempt to identify functional resistance genes for future hereditary rearing projects in grass carp.

**Results:**

This study used ENU-mutated grass carp to identify genetic markers associated with resistance to the haemorrhagic disease caused by GCRV. Bulked segregant analysis (BSA) was performed on two homozygous gynogenetic ENU grass carp groups who were susceptible or resistant to GCRV. This analysis identified 466,162 SNPs and 197,644 InDels within the genomes of these mixed pools with a total of 170 genes annotated in the associated region, including 49 genes with non-synonymous mutations at SNP sites and 25 genes with frame shift mutations at InDel sites. Of these 170 mutated genes, 5 randomly selected immune-related genes were shown to be more strongly expressed in the resistant group as compared to the susceptible animals. In addition, we found that one immune-related gene, *EPHB2*, presented with two heterozygous SNP mutations which altered the animal’s responded to GCRV disease. These SNPs were found in the intron region of *EPHB2* at positions 5859 (5859^G > A^) and 5968 (5968^G > A^) and were significantly (*p* = 0.002, 0.003) associated with resistance to GCRV. These SNP sites were also shown to correlate with the GCRV-resistant phenotype in these ENU grass carp. We also evaluated the mortality of the different ENU fish genotypes in response to GCRV and the SNPs in *EPHB2*. The outcomes of these evaluations will be useful in future selections of GCRV-resistant genes for genetic breeding in grass carp.

**Conclusion:**

Our results provide a proof of concept for the application of BSA-sequence analysis in detecting genes responsible for specific functional genotypes and may help to develop better methods for marker-assisted selection, especially for disease resistance in response to GCRV.

**Supplementary Information:**

The online version contains supplementary material available at 10.1186/s12864-021-07858-x.

## Background

Genetic breeding of aquaculture fish species primarily depends on the identification of naturally occurring mutants with high performance, and subsequent hybridisation or marker-assisted breeding to produce better-quality strains [1]. These methods have been used to produce more than 200 improved strains of aquaculture fish species in China, with 139 produced using genetic selection. Therefore, chemical mutagenesis is an effective way to make new mutants for upcoming genetic development in aquaculture species [2]**.** *N*-ethyl-*N*-nitrosourea (ENU) is a chemical mutagen that acts as an alkylating agent, exchanging its ethyl gather to nucleophilic nitrogen or oxygen locales on the deoxyribonucleotides, leading to base inconsistencies during DNA replication [3, 4]. ENU mutagenesis has been demonstrated to be effective for inciting point mutations in the genome of the grass carp [5].

Grass carp reovirus (GCRV) belongs to the Reoviridae family genus Aquareovirus. The first instance of haemorrhagic disease in grass carp was reported at a fish farm in Hubei Province in 1972 [6]. GCRV was categorised as an aqua reovirus and in 1984 its genome was shown to include 11 sections of double stranded RNA. The Aquareovirus genus can be separated into groups A through G (AQRV-A to AQRV-G), with GCRV falling into the AQRV-C group [7]. GCRV infection results in haemorrhagic disease in grass carp and causes noteworthy losses in fingerlings, with recent outbreaks exhibiting a significant economic impact.

A genotyping-by-sequencing method, permits genome widespread association studies, bulked segregant analysis (BSA) and genomic selection and has previously been applied to various animal breeding programmes. Recent reductions in the cost of genome sequencing have opened up the opportunity for whole genome sequencing and resequencing in larger pools of individuals. BSA has been suggested as an effective tool for quickly identifying markers connected to specific characteristics of interest including those associated with disease resistance [8]. This approach includes segregating the F2 population produced from a starting cross of two phenotypically different parents, which are then scored for the phenotype of interest. Bulked DNA or RNA tests are built from organisms with differentiating phenotypes. BSA has been primarily used in the development and identification of crop species as it facilitates the recognisable identification of large impact QTLs, such as disease resistance genes or for mapping subjective mutations [8–11].

The objective of the current study was to select the functional genotypes from ENU fish facilitating GCRV resistance using BSA sequence analysis. Our study also provides a useful method for carrying out marker-assisted selection, especially for disease resistance in response to GCRV.

## Results

### SNP and InDel annotation

**Fig. 1:**
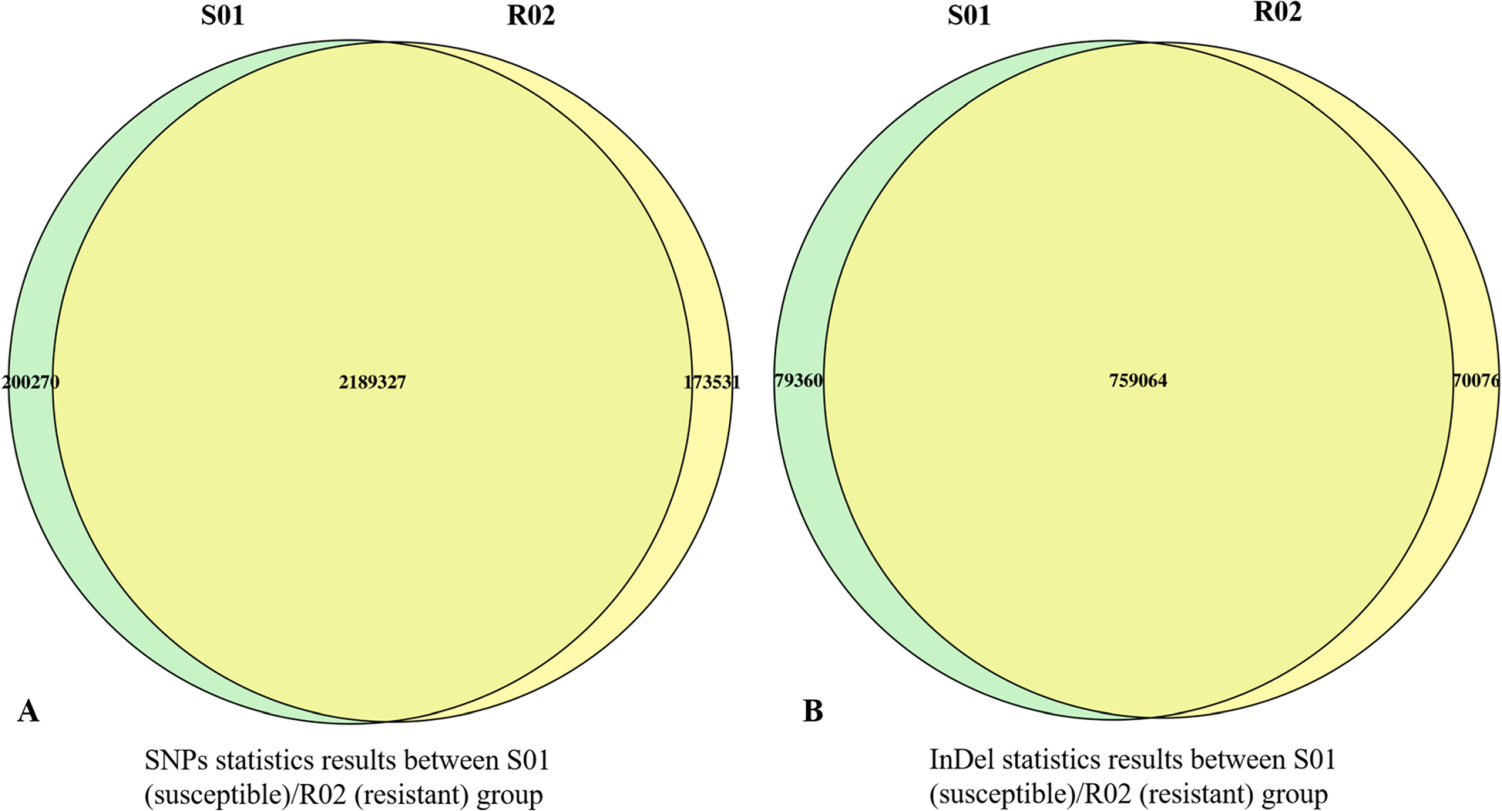
Venn diagram of SNP/ InDel statistics between sample: Venn diagram describes the overlapped pathways between resistant and susceptible groups. (A) SNPs statistics results between S01 (susceptible)/R02 (resistant) group, (B) InDel statistics results between S01 (susceptible)/R02 (resistant) group

After filtering our sequencing analysis produced 73.47 Gbp of clean read data with an average Q30 of 93.92%, and an average GC% of up to 38.11% (Table 1). The average genome coverage depth in each sample was 41.00X, and the genome coverage was about 99.11% (at least 1×) **(**Table 1**)**, indicating the high quality of the sequencing data. The assembled genome size of grass carp is 900.50 Mb, GC content is 37.42% and the genome is currently annotated at Scaffold level [12]. The Venn diagram reveals that there are a different number of SNP and InDels in the S01(susceptible) and R02 (resistant) groups (Fig. 1). There were approximately 200,270 SNPs in the S01 and 173,531 SNPs in the R02 groups. The overlapping portion of the Venn diagram represented the total number of SNPs (2,189,327) and InDels (759,064), identified in these assays (Fig. 1).

**Table 1:** Summary of Illumina sequencing data

Sample ID	Susceptible group (S01)	Resistant group (R02)	Average values
Clean_reads	125,577,407	117,707,723	73.47Gbp
Q30 (%)	93.52	94.31	93.92%,
GC (%)	38.09	38.14	38.11%
Mapped ratio (%)	80.51	80.53	80.52%
Average depth	42	40	41.00X
Coverage_ratio_1× (%)	99.17	99.05	99.11%
Coverage_ratio_5× (%)	97.77	97.57	97.67
Coverage_ratio_10× (%	96.26	95.97	96.12

We detected a total of 466,162, 197,644, 8492, and 3668 polymorphic sites across different regions of the genome using SNP, InDel, and SNP/InDel annotation (Table 2).

**Table 2:** Annotation results statistics of SNPs and indels containing candidate region

Different region and types of Mutation	SNP annotation result statistics	InDel annotation result statistics	SNP annotation results Statistics in candidate regions	InDel annotation results statistics in the candidate regions
	**S01vsR02**	**S01vsR02**	**S01vsR02**	**S01vsR02**
INTERGENIC	220,527	89,734	4223	1797
INTRON	144,560	27	2538	1242
UPSTREAM	37,653	66,109	618	266
DOWNSTREAM	37,132	16,283	653	232
UTR_5_PRIME	3202	16,315	43	25
UTR_3_PRIME	4074	1945	60	26
SPLICE_SITE_ACCEPTOR	118	2169	1	1
SPLICE_SITE_DONOR	100	94	2	2
SPLICE_SITE_REGION	912	66	20	5
FRAME_SHIFT	–	–	–	50
CODON_INSERTION	–	–	–	7
CODON_DELETION	–	–	–	6
CODON_CHANGE_PLUS_CODON_INSERTION	–	–	–	3
CODON_CHANGE_PLUS_CODON_DELETION	–	–	–	3
START_GAINED	648	401	7	–
START_LOST	5	15	–	–
SYNONYMOUS_START	3	3272	1	–
NON_SYNONYMOUS_START	1	276	–	–
SYNONYMOUS_CODING	7251	271	139	–
NON_SYNONYMOUS_CODING	9298	150	180	–
SYNONYMOUS_STOP	71	277	–	–
STOP_GAINED	449	178	6	2
STOP_LOST	158	62	1	1
Other	0	0	0	0
Total	466,162	197,644	8492	3668

These annotation were then used to demonstrate that the SNPs were randomly distributed throughout the genome **(**Table 2**),** while the InDel frameshift mutations were found in only 50 genes, 7 of which demonstrated an in codon_insertion and 6 of which had an in codon_deletion **(**Table 2**).** Synonymous and non-synonymous codons had a higher number of SNPs than other genes (7251, 9298) **(**Table 2**)**.

### Association analysis (SNP, InDel) and ED correlations

The SNPs and InDels were first filtered leaving 2,313,014 high-quality trusted SNP sites and 775,981 high-quality InDels which were then applied to the association analysis **(**Table 3**)**. Through this, 1197 Scaffold/contig sequences with significantly enriched associated SNP sites were selected. Some results are shown in the table **(**Additional file 1**).** The same analysis method (Euclidean Distance (ED) method) as the SNP association analysis was used, and 85 Scaffold/contig sequences that were significantly enriched in association InDel sites were finally screened **(**Additional file 2). Take the intersection of the results obtained by these two association analysis methods (SNP & InDel along with ED), a total of 21 Scaffolds related to traits were obtained (Table 4**).**

**Table 3:** SNP and InDel filtering statistics

Filtering statistics	Total SNP	Total InDel	Multiple allele loci	Read support for sites less than 4	Loci with consistent pool	High quality number after filteration
SNP	2,563,128	**–**	2212	40,627	207,275	2,313,014
InDel	**–**	908,500	36,042	37,050	59,427	775,981

**Table 4:** Candidate Scaffold results

Scaffold	AllSNP	AssoSNP_ED	AssoInDel_ED
CI01000352	2484	306	85
CI01112186	5	3	4
CI01000136	2141	185	73
CI01000184	1334	121	51
CI01000190	2631	214	83
CI01000087	2767	286	84
CI01000240	274	50	18
CI01061811	5	2	3
CI01072320	5	7	3
CI01163490	5	1	3
CI01064712	6	2	3
CI01085134	23	10	5
CI01075334	3	4	2
CI01141415	3	3	2
CI01000257	280	30	16
CI01079816	4	2	2
CI01114229	4	2	2
CI01017852	12	4	3
CI01026461	5	3	2
CI01114140	5	3	2
CI01000262	112	33	8

### Gene annotation and functional analysis

**Fig. 2:**
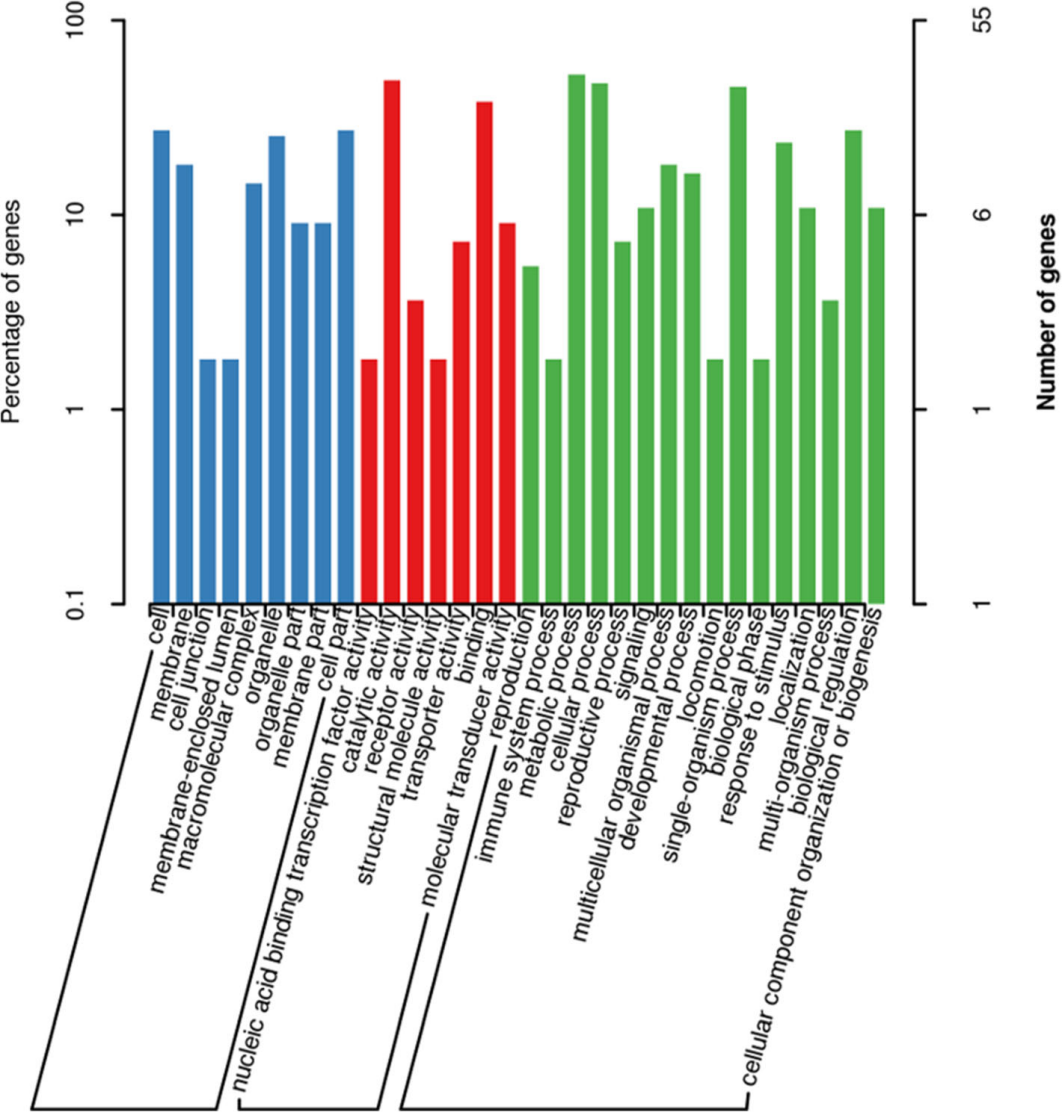
Clustering map of GO in candidate region: The abscissa is the content of each category of GO, the left of the ordinate is the percentage of the number of genes, and the right is the number of genes. This figure shows the gene classification of each secondary function of GO in the context of all genes in the associated region

**Fig. 3:**
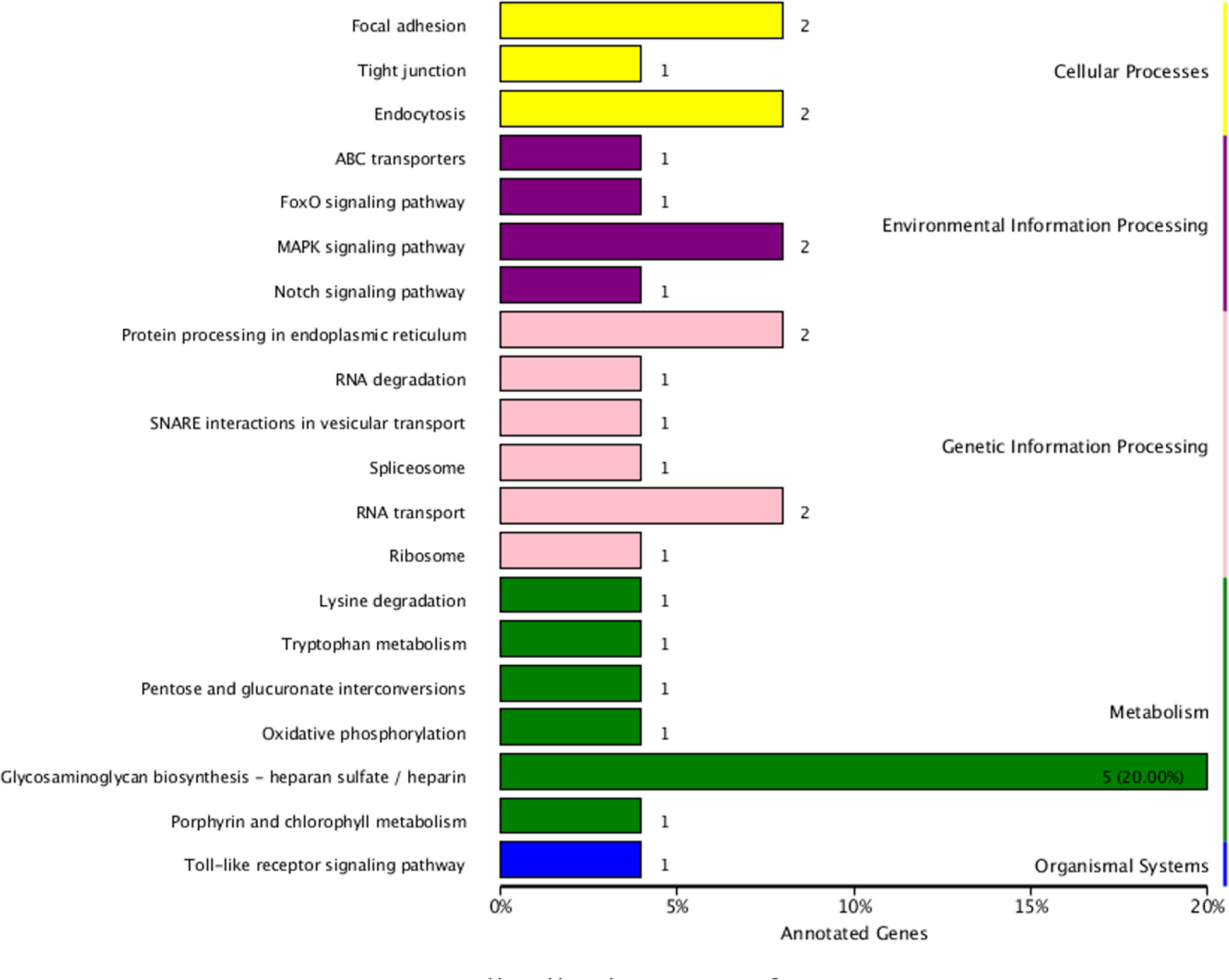
KEGG distribution map of genes: Top 20 KEGG pathways of genes containing SNPs associated with resistance to GCRV in ENU. The number of SNPs is shown behind the corresponding pathways

**Fig. 4:**
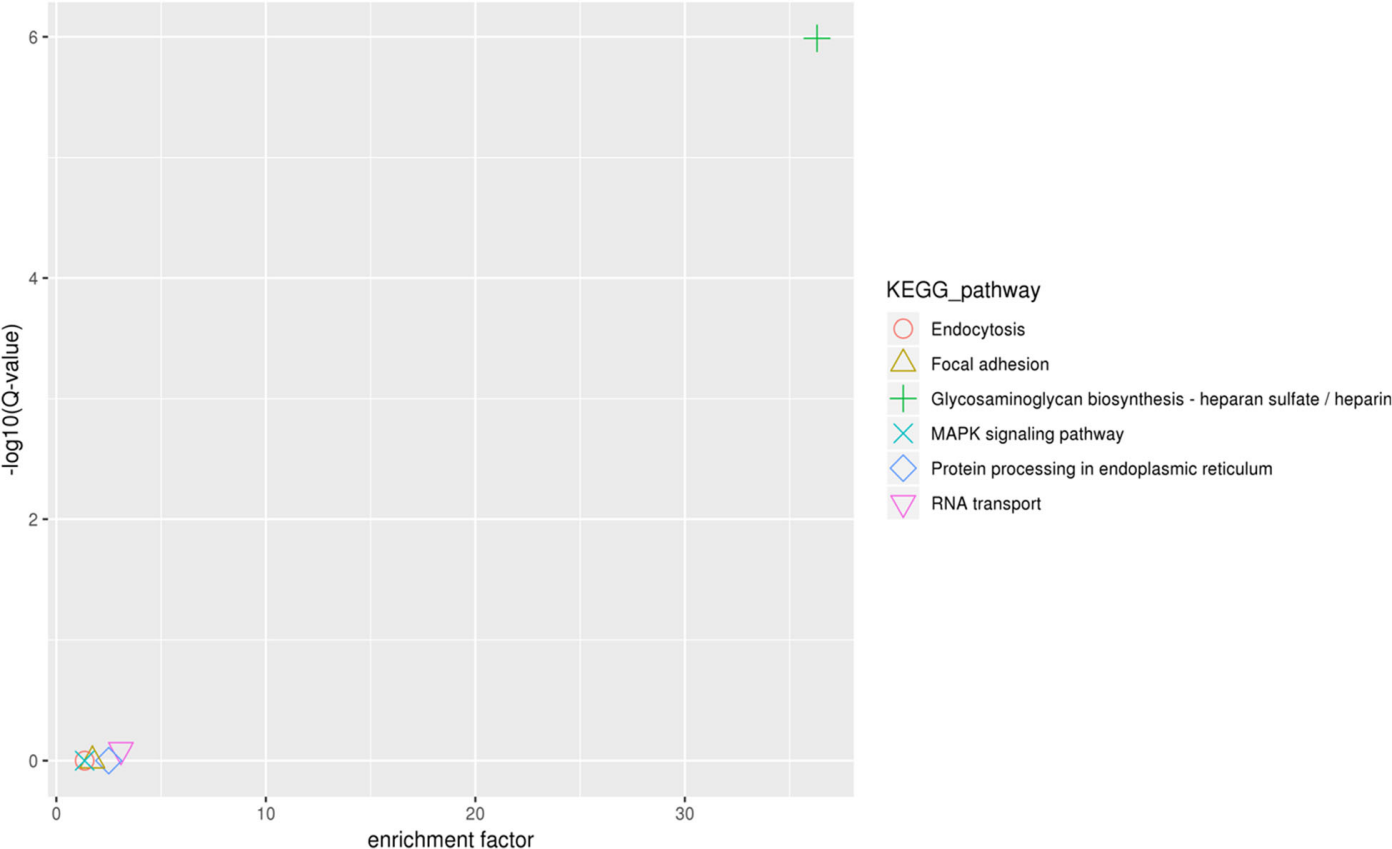
KEGG enrichment of genes in candidate regions: The enrichment pathways with more enrichment factor and significance values were “Glycosaminoglycan biosynthesis-heparan sulfate / heparin (ko00534)”, “RNA transport (ko03013)”, “Protein processing in endoplasmic reticulum (ko04141)”, “Focal adhesion (ko04510)”, “Endocytosis (ko04144)”, and “MAPK signaling pathway (ko04010)”

A total of 170 genes were identified within the candidate region, 49 of which had non-synonymous mutations and 25 of which presentenced with frameshift mutation within the mixed pool evaluations **(**Table 5**,** Additional file 3**)**. A total of 55 genes in the genome were annotated and classified into biological processes, cellular components, and molecular functions **(**Fig. 2**,** Additional files 4, 5 & 6**).** An additional 11 genes were found to have non-synonymous mutations and 2 other genes were found to have frameshift mutations when evaluated for GO enrichment. The annotated KEGG databases showed that 29 SNPs were found in the top 20 pathways as shown in Fig. 3 and after multiple-testing corrections, we selected those pathways with Q values of ≤ 0.05 as significantly enriched for these genes **(**Fig. 4**)**. Our data suggest that these genes may play an important role in the innate immune response to GCRV in ENU grass crap.

**Table 5:** Statistics of gene function annotation results in SNP and InDel in candidate regions

Annotated_databases	Gene Num	Non_Syn Gene Num	FRAME_SHIFT Gene Num
NR	159	46	25
NT	170	49	25
trEMBL	170	49	25
SwissProt	104	30	19
GO	55	11	2
KEGG	69	13	7
COG	35	11	6
Total	170	49	25

### Changes in gene expression associated with grass carp reovirus (GCRV) resistance

**Fig. 5:**
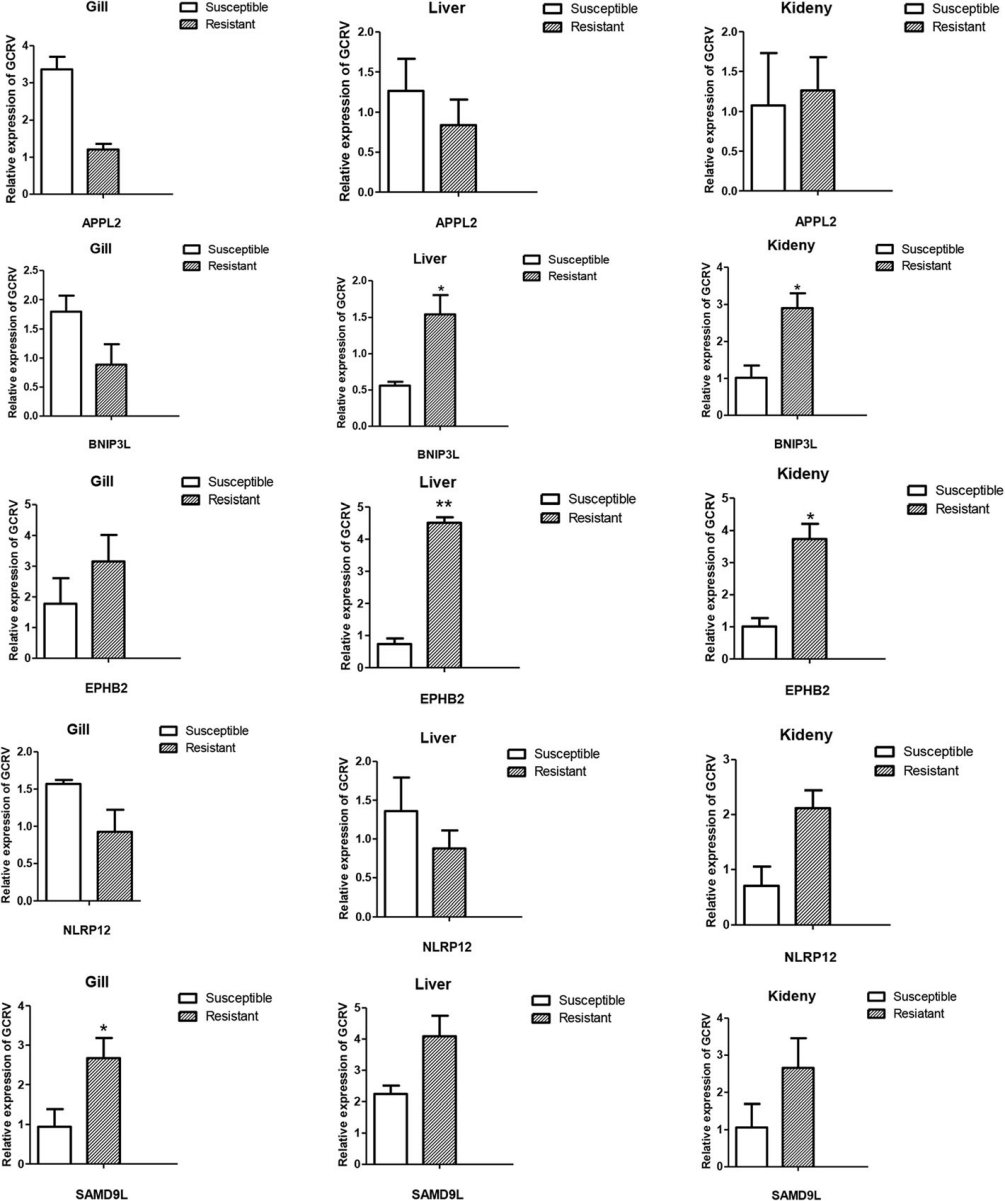
genes expression analysis showed that mRNA expression level of SAMD9L, BNIP3L and *EPHB2* was highly significant expressed in resistant group than infected group in response of GCRV (*p* < 0.01), APPL2 expression level was higher in liver and kidney as compared to gill tissue in case of resistant group. Kidney-NLRP12 gene expression level was significantly highly expressed in resistant group as compared to infected group after GCRV infection (*p* < 0.01). The statistical results (expressed as mean ± standard deviation) were analyzed by one-way analysis of variance, followed by Dunnett’s test for multiple comparisons using IBM SPSS Statistics 22 software. *p* < 0.01, *p* < 0.05 was considered to be statistically significant

The relative expression levels of the five genes significantly associated with grass carp reovirus (GCRV) resistance were evaluated using *q*PCR **(**Fig. 5**)**. We selected five genes with genetic variations and examined their mRNA expression levels in liver, kidney and gill tissues. Each of the five genes were shown to be involved in the inflammatory response, cell proliferation, anti-apoptosis, tumour suppression and the immune response to viral infection pathways. Results showed that mRNA expression levels of SAMD9L, BNIP3L and *EPHB2* were significantly increased in the resistant group when compared to the susceptible group (*p* < 0.01), APPL2 expression was higher in the liver and kidney when compared to the gill tissues of resistant GCRV resistant fish. Kidney-NLRP12 gene expression was significantly elevated in the resistant group when compared to the susceptible group (*p* < 0.01) **(**Fig. 5**)**.

### Verification of SNPs associated with grass carp reovirus (GCRV) resistance

**Fig. 6:**
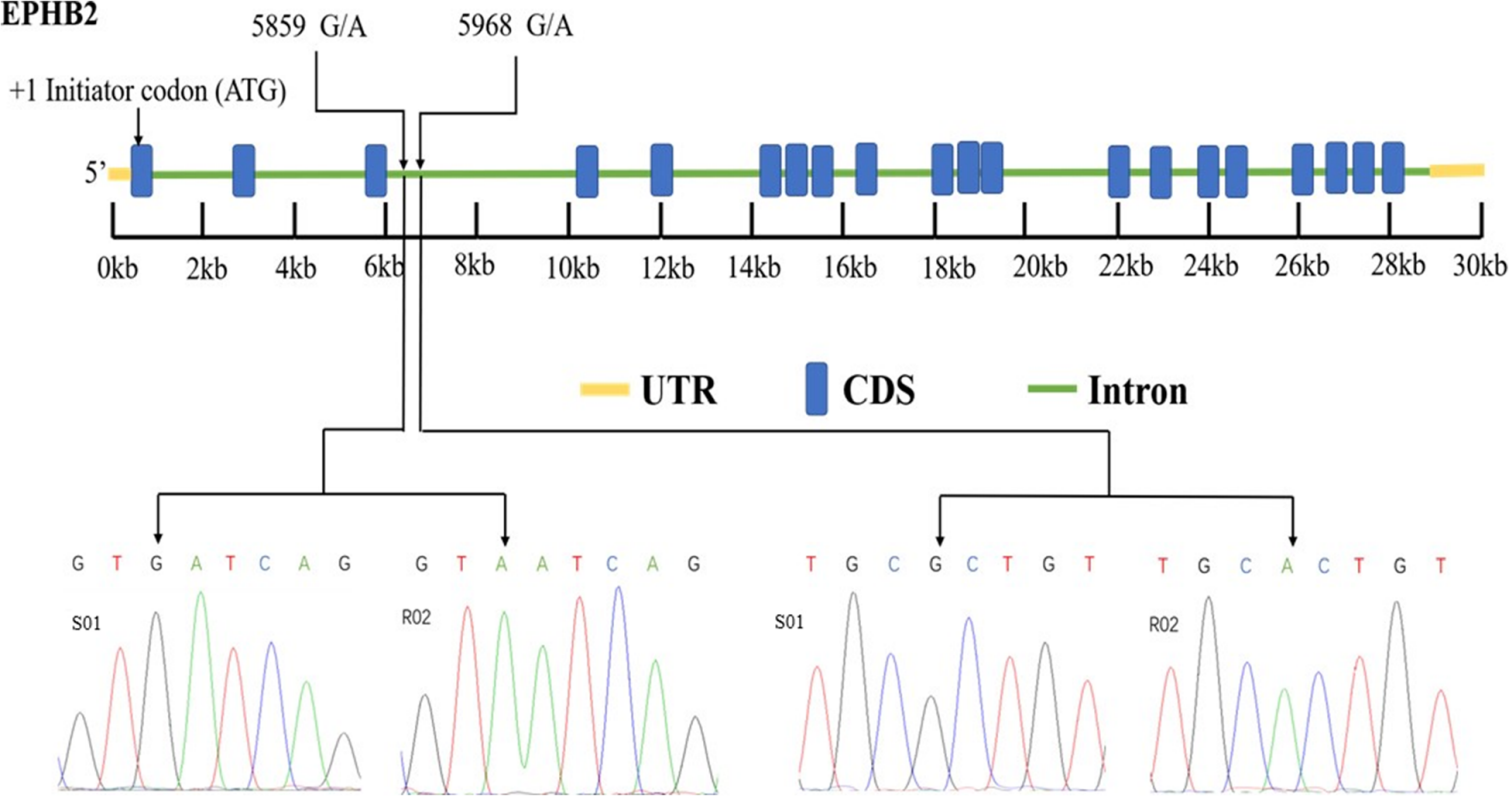
Genomic structure and SNP confirmation sites of *EPHB2* gene in ENU: 5’UTR start site was regarded as the + 1-initiator codon (ATG) in *EPHB2* shown with blue colour (CDS). In *EPHB2*, SNPs were found out in 2 position which has been mentioned with chromosomal position 5859, 5968 determine a GA mutation in S01 (susceptible)/R02 (resistant) group. Green colour shows intron region

After validation, we identified an SNP (CI01000190: 1067676 A > G) at the 1,067,676 position and another SNP (CI01000190: 1067785 A > G) at 1067785 in chromosome CI01000190 **(**Table 6**).** Both SNPs were located in the intron region of *EPHB2* as shown in Fig.6. SNPs in *EPHB2* were found in two positions as described above and were shown to encode a 5859^G > A^ and 5968^G > A^ mutation in the S01/R02 groups.

**Table 6:** Genes with validated SNP

Chromosome	Gene	SNP position	Ref	S01	R02	ED-value
CI01000190_01061817_01090001.mRNA	EPHB2	1,067,676	R	R	R	0.624991
		1,067,785	R	R	R	0.648181

About 300 ENU fish were used for amplification and sequencing of SNP sites across the *EPHB2* gene. The confirmed *EPHB2* SNPs are summarised in Table 7 for both the resistant (R02) and susceptible (S01) groups. In the case of the first SNP, the allele frequencies of the A and G in surviving fish (resistant) were 24 and 76%, respectively, while in the dead fish (susceptible), the allele frequencies of A and G were 66 and 34%, respectively. At the other SNP, the allele frequencies of A and G in the surviving fish were 18 and 82% whereas in the dead fish they were 62 and 38%, respectively **(**Table 7**)**. The chi-squared test showed that the allele frequencies were significantly different between the dead and surviving animals (*p* = 0.002 and *p* = 0.003 for respective SNPs) **(**Table 7**)**, suggesting that *EPHB2* is closely associated with GCRV resistance.

**Table 7:** Number and allele frequency at two SNPs of EPHB2 in susceptible and resistant ENU after GCRV infection

SNP1(CI01000190, 1,067,676) (*p* = 0.002)	Allele frequency in resistant group (*n* = 50)	Allele frequency in susceptible group (*n* = 50)
A	24%	66%
G	76%	34%
SNP2(CI01000190, 1,067,785) (*p* = 0.003)		
A	18%	62%
G	82%	38%
SNP1 genotype (*p* = 0.004)	Allele frequency in resistant group (*n* = 50)	Allele frequency in susceptible (n = 50)
AA	16%	34%
AG	48%	52%
GG	36%	14%
SNP2 genotype (*p* = 0.003)		
AA	12%	30%
AG	44%	52%
GG	44%	18%

**Fig. 7:**
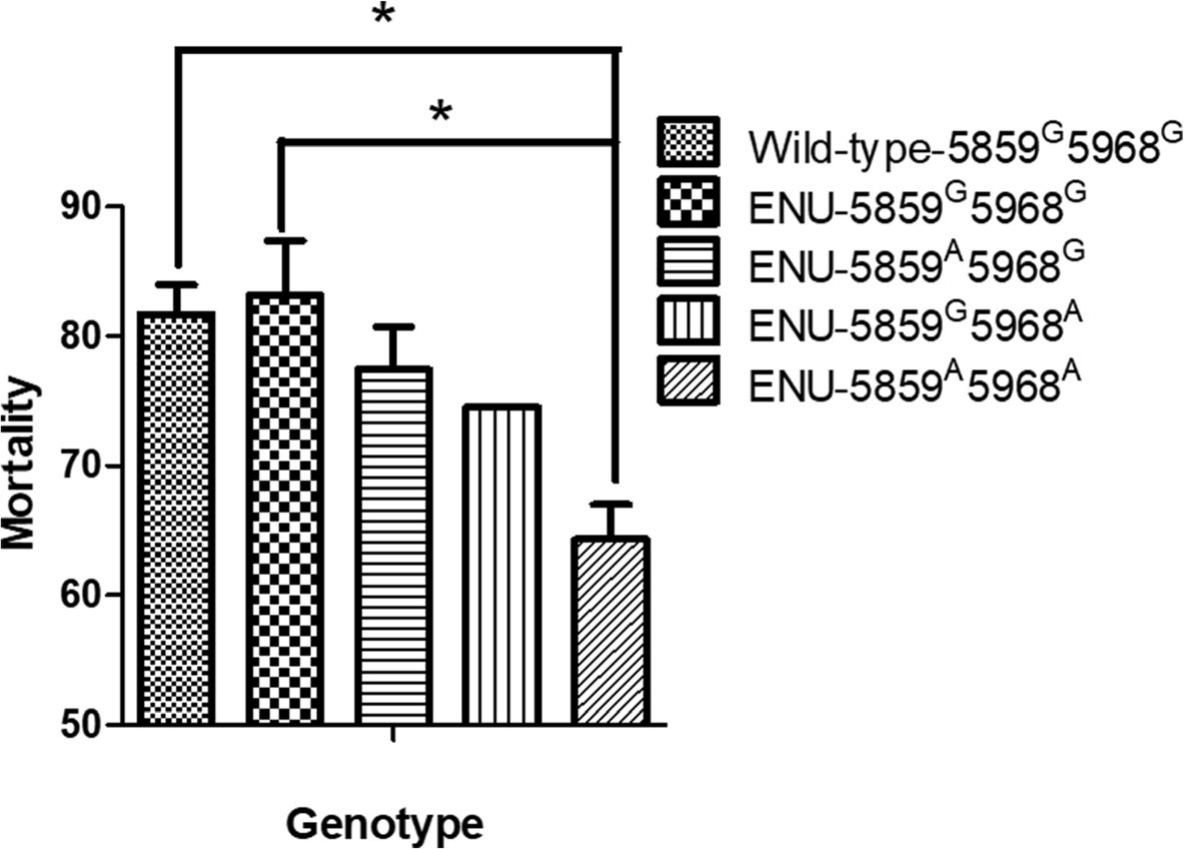
Wild type and mutant grass verification on the basis of different SNP genotype and mortality rate

This SNP sequencing data was then used to place the ENU fish into four groups according to their genotype **(**Fig. 7**):** Group I 5859^G^ 5968^G^, Group II 5859^A^ 5968^G^, Group III 5859^G^ 5968^A^ and Group IV 5859^A^ 5968^A^ **(**Fig. 7**).** We then evaluated the correlation between each genotype and GCRV resistance. The results show that resistance was related to genotype. The mortality rate in Groups II and III were 77.4 and 74.5%, which were lower than Group I. However, the mortality rate in Group IV was the lowest (64.3%) and significantly lower than that in Group I. Our previous research suggests that wild-type grass carp mortality is 81.68% (SNP screening showed that the genotype of the wild-type grass carp was 5859^G^ 5968^G^) which indicates that there was a similar resistance in the wild-type and Group I grass carp subjects.

## Discussion

Association analysis remains the key method for mining disease resistance SNPs in marine animals. Previous work has reported that SNP frequencies differ between different populations in numerous species [13–15]. However, to the best of our knowledge, there is limited information related to immune associated SNPs and their impact of GCRV resistance in grass carp. Therefore, the currently study focused on the selection of functional genes and their SNPs in relation to GCRV resistance in ENU grass carp, in the hope of improving our understanding of GCRV resistance and establishing a method to facilitate the selection of disease-resistant strains of ENU grass carp in culture.

Cultured grass carp are highly vulnerable to diseases which lead to yield reduction, and while antibiotics and drugs have been used to control the problem the regular use of these alternatives has various unwanted side effects on both the animals and the environment. Therefore, disease-resistant grass carp are highly desirable as they can greatly increase fish yield [5]. Due to the low occurrence of natural mutations in grass carp, chemical mutagenesis can be useful for increasing genetic mutation rates [2]. In this study, homozygous gynogenetic ENU grass carp, which have the advantage of strong disease resistance and rapid growth, were used to determine the functional genotypes associated with GCRV resistance using BSA sequence analysis.

We used BSA technology to determine SNP mutations within the genomic regions associated with GCRV resistance in homozygous gynogenetic ENU grass carp. Our findings verified the viability of using a BSA approach and the extensive information available for SNP typing for disease resistance traits as a fast and affordable method for marker development. Zhang et al (2019) also demonstrated that genetic analysis using BSA-sequencing is effective for accelerating the identification of disease resistance markers and will assist the selective breeding of turbot resistant to *Vibrio anguillarum* [16].

SNP and InDels are used for annotating mutations and predicting the effects of these mutations [17]. SNP mutation is a significant source of genetic diversity facilitating molecular evolution and disease resistance. Some researchers focus on non-synonymous coding SNPs, because those SNPs might directly influence protein activity [18]. Wang et al (2015) reported the significant differences in both SNP and InDel rates in resistant and susceptible *C. idella* genomes. However, the SNPs and InDels associated with resistance/susceptibility to GCRV were not described in this omics sequencing project [12]. SNPs can provide innovative resources for genome sequence modification and facilitate the study of selective breeding [19]. This study identified a total of 466,162 SNPs and 197,644 InDel in the resistant/susceptible group, but only 9298 SNPs caused non-synonymous mutations.

In current study, there are indication of several resistance genes in the S01/R02 group of ENU grass carp. These were then evaluated by GO classification and KEGG pathway analysis to identify the molecular functions of the candidate genes in the resistant group. The correlation of differentially expressed sequences with the whole sequences of equivalent GO groups or KEGG pathways was observed as the key measure of enrichment factor [20, 21]. Our results showed that the KEGG pathways with SNP enrichment ratio were binding in resistant groups. GO analysis also showed that some cellular components tended to be less polymorphic than others, whereas KEGG pathway analysis showed that some pathways tended to be more polymorphic than others. SNPs are inequitably distributed across the genome with many associated with higher contingency in functionally important positions. Similarly, SNPs can affect the components and their interactions [22]. These clarifications should be investigated in future studies.

Gene mutations may introduce phenotypic variation by influencing gene expression, including the possibility of hybrid vigour as useful traits that are oppressed in animal and crop breeding [23]. There has long been a tacit understanding that gene expression differences play a vital role in species differentiation and experiments on natural selection for gene expression can now be monitored in more effective ways [19, 24]. In our analysis, we selected 5 genes with genetic variations and examined their mRNA expression in liver, kidney and gill tissues. The results of these analyses showed that mRNA expression levels of SAMD9L and *EPHB2* were increased in all three tissues from the resistant groups in response to GCRV, while BNIP3L and APPL2 expression was increased in the liver and kidney tissues of resistant fish when compared to the gill tissues of the same animal. Kidney-NLRP12 gene expression was higher in the resistant group as compared to susceptible group and the results of the expression analysis of all five of these genes suggest that each may be partially responsible for GCRV resistance.

Several more recent studies have found that SNPs in intron and intergenic regions also play a significant role in the adaptation of specific traits. It was reported that, SNPs in the third intron of the F-box and leucine rich repeat protein 17 (FBXL17) gene explains 58.4% of the phenotypic differences in sex reversal of Chinese tongue sole *Cynoglossus semilaevis* [25]. Li, et al. (2016), also reported that heterozygous SNP variation can contribute to increase latex yield in these hybrids [26]. Here, we identified two heterozygous SNPs (at chromosomal position 5859G > A and 5968G > A) in the intron region of *EPHB2* which were shown to be significantly associated with GCRV responses in resistance/ susceptible ENU grass carp. Allele G was higher in the resistant group than in the susceptible group, indicating that these alleles could increase disease resistance. The survival rate of susceptible ENU after GCRV infection might be reduced by higher frequencies of allele A at these SNPs. Consequently, future breeding programmes for these ENU grass carp could emphasise selection for the G allele at both of these SNPs to improve GCRV disease resistance. This study is, to the best of our knowledge, the first to report SNPs located in a gene that is associated with GCRV disease resistance in ENU grass carp. Additionally, the SNPs obtained in this study provide the basis for genetic selection of GCRV disease resistance in ENU grass carp.

*EPHB2* (Ephrin type-B receptor 2) influences the immune system in numerous ways, primarily through immune cell transfer and activation. Regulation of B and T lymphocyte as well as dendritic cell activation has all been linked to *EPHB2* [27, 28]. The expression of *EPHB2* can also be regulated by certain inflammatory cytokines and pathogen-related molecular forms [29, 30]. Among the five resistant genes studied in this paper, we only found SNPs in *EPHB2* as explained above, and this gene was highly expressed in the resistantt group. The modification and higher expression of this gene indicate that it is closely associated with GCRV resistance in these animals. This gene functions as a tumour suppressor and plays a role in immune cell enhancement during GCRV disease in ENU grass carp. Our results concluded that *EPHB2* is involved in the immune response and that it can repress viral replication and attenuate acute inflammatory responses to protect cells.

## Conclusions

Our results demonstrate the utility of applying BSA-sequence analysis to the detection of genes responsible for interesting phenotypes and will help in developing new protocols for completing marker-assisted selection for GCRV disease resistance in farmed fish. *EPHB2* expression was higher in the kidney, liver and gills. Two SNPs found in the intron region of the *EPHB2* gene were significantly associated with GCRV resistance. Additionally, *EPHB2* is involved in immune response and may suppress virus replication and reduce acute inflammatory responses protecting cells from apoptosis. Taken together these data suggest that *EPHB2* may be an important gene for GCRV resistance. The SNPs associated with GCRV resistance could be applied to marker-assisted selection for breeding GCRV resistant grass carp.

## Materials and methods

### Experimental design

ENU mutant grass carp (meiotic gynogenetic offspring induced by UV inactivated heterologous sperm from *Megalobrama amblycephala*) were obtained from the Bream Genetics and Breeding Center at Shanghai Ocean University, Shanghai, China. On arrival to the laboratory, fish were maintained at 28 ± 0.5 °C for at least 7 days prior to experimental use and fed well to make them as healthy as possible. We used a total of 200 fish with an average weight of 4.4–6.0 g. The trials were conducted in aerated glass aquariums (120 × 40 × 30 cm) each containing 100 L of water. After acclimatisation, all fish were intraperitoneally inoculated with 20 μl/g of GCRV-873. Fish started to die 9 days post infection and 30 fish were collected from this group (susceptible) and identified by their extreme GCRV symptoms. At 14 days post infection any surviving fish (30 animals) were collected and classified as resistant for further evaluation.

### Fish sampling

Using the method described above we selected two groups of ENU fish with extremely different phenotypes. A total of 30 fish were selected as the susceptible/ infected/morbid group (S01) and 30 fish were selected as the resistant group (R02). Infection was allowed to run for 14 days before we collected liver tissue samples from each group and applied these to the BSA analysis.

### Sequencing analysis

Genomic DNA was isolated using a conventional phenol-chloroform extraction strategy in combination with RNase treatment and stored at − 20 °C until use. Two bulk isolations were produced by pooling an equal quantity of DNA from a susceptible (S01) and a resistant group (R02). DNA from each pool was then used to build paired-end (PE) sequencing libraries, which were sequenced on an Illumina HiSeq (Illumina Casava 1.8 version). The entire experimental procedure was completed as described in the protocol form Illumina, including sample testing, library construction, library-quality testing, and computer sequencing. The final sequence read length was 150 bp (Biomarker technology, Beijing, China).

After removing the connector sequences and low-quality reads, the clean reads were then re-evaluated for quality utilising FASTQC. High quality PE reads were mapped to the grass carp reference genome sequence (PRJEB5920) [12] using the BWA programme with default constraints [31]. The position of the clean reads on the reference genome was identified by comparing data such as the sequencing depth and genome coverage in each run and this was then used to map the mutation loci. Assessments of the sequencing output information, comparisons, depth of coverage, and the genome coverage comparison at each depth for both the S01 and R02 groups are summarised in Table 1**.**

### SNP and InDel detection

SnpEff is a software package designed to facilitate the annotation of mutations (SNP, Small InDel) and predicting the effects of these mutations [17]. The detection of SNPs was mainly implemented using the GATK software toolkit [32] and Picard (http://sourceforge.net/projects/picard/) was used to complete the data preprocessing such as marking the duplicates. GATK was used for Local realignment and base recalibration to ensure detection.

InDel represents single base insertions and deletions, with the insertions detected using GATK. Small InDel variation is generally less frequent than SNP variation, and is also reflected in the differences between the sample and the reference genome, and InDels in the coding region will cause frameshift mutations, resulting in changes in gene function.

### ED calculations

ED algorithm uses sequencing data to identify markers which demonstrate significant differences in occurrence between pools and is also often used to evaluate SNP/InDel associations [8]. Theoretically, two mixed pools constructed using the BSA system are likely to have differences in the target trait-related sites, but the other sites should be consistent, so the ED value of non-target sites should tend towards 0. The formula for the ED calculations is shown below. The larger the ED value, the greater the difference between the mark and the two mixing tanks.

$$ ED=\sqrt{{\left({A}_{mut}-{A}_{wt}\right)}^2+\kern0.5em {\left({C}_{mut}-{C}_{wt}\right)}^2+\kern0.5em {\left({G}_{mut}-{G}_{wt}\right)}^2+\kern0.5em {\left({T}_{mut}-{T}_{wt}\right)}^2} $$

where each letter (A, C, G, T) corresponds to the frequency of its corresponding nucleotide in the mutation and wild type pool or bulk preparation respectively.

### Functional annotation of genes containing SNPs

Those genes with SNPs correlated to the resistance/susceptibility to GCRV were annotated using BLAST software against multiple databases including the NR [non-redundant protein database, NCBI], Swiss-Prot [http://www.uniprot.org/], GO [Gene Ontology, http://www.geneontology.org/], KEGG [http://www.genome.jp/kegg/] [33, 34], COG [http://www.ncbi.nlm.nih.gov/COG/]) coding genes in the candidate interval. This in-depth annotation allowed for rapid screening of candidate genes for functional relevance.

### Gene expression and SNP verification

*q*PCR was carried out on a CFX96 Touch™ Real-Time PCR System, using SYBR Premix Ex Taq kit (TaKaRa, Japan). All primers were designed using Primer Premier 5.0 software and are listed in Table 8. The comparative expression values of each of the designated genes versus 18 s rRNA (reference gene to normalise expression levels between samples) was calculated using the 2^−∆∆Ct^ method. SYBR Green reactions were performed in 20 μL volumes containing 10 μL of 2 × SYBR® Green Realtime PCR Master Mix (Toyobo, Osaka, Japan), 1 μL of each forward and reverse primer (10 μM), 7 μL of water, and 1 μL of diluted cDNA (100 ng/μL). All experiments were performed in two groups. We identified five functional genes as disease-related mutations in response to GCRV in mutant grass carp.

**Table 8:** Selected primer and sequence

Gene	Primer name	Forward primer (5′-3′)	Reverse primer (5′-3′)	Temperature
CI01000184_00984445_00984988.mRNA	APPL2	CCGTGGATGACACCGCCTAC	CACACTCCACGGCCATGACA	59.7
CI01000087_01389941_01395452.mRNA	BNIP3L	TCTGAGTGTGTCCATCGAGT	GAGATCCAGCAGAACACTGG	58.0
CI01000190_01446777_01465687.mRNA	NLRP12	TCTGAGTGTGTCCATCGAGT	GAGATCCAGCAGAACACTGG	58.0
CI01000190_01061817_01090001.mRNA	EPHB2	CAGAGGCATTCATCTTCCCA	GAGAGAGGGCAGGGGAAAA	58.1
CI01000240_00049921_00050822. mRNA	SAMD9L	GCAGCAGAATGATGTGGTGAC	TGTGTTGGTGAATGCTTTGAAT	58.5

For the SNP verification, we injected GCRV into 300 ENU grass carp and selected 50 dead mutant fish on 8-day and 50 alive mutant fish on day 14 for allelic frequency evaluations (Table 8**)**. Both SNPs were located in the intron region of *EPHB2*. We further verified these SNPS in our previous data set in which the fish were divided into 4 group on the basis of their genotype and compared their mortality rate with the wild type grass carp after challenge with GCRV. We also evaluated the statistical association between each genotype and resistance, using the *p*-values produced from a chi-square test. A subsequent *p*-value of 0.05 or less was determined to be statistically significant**.**

### Statistical analysis

Statistical significance (expressed as mean ± standard deviation) was evaluated using one-way analysis of variance, followed by a Dunnett’s test for multiple comparisons using IBM SPSS Statistics 22 software. *p* < 0.01 and *p* < 0.05 were considered statistically significant. All experiments were repeated at least three times.

## Supplementary Information

**Additional file 1.**

**Additional file 2.**

**Additional file 3.**

**Additional file 4.**

**Additional file 5.**

**Additional file 6.**

## Data Availability

Raw genomic-seq reads data supporting the results of this article are available in the NCBI Sequence Read Archive (SRA) database (Acession number: PRJNA716293).

## Abbreviations

BSA: Bulk segregant analysis
SNP: Single nucleotide polymorphism
InDel: Insertion/ deletion
ENU: Mutant grass carp
GCRV: Grass carp reovirus
GATK: Genomic analysis toolkit
SnpEff: SNP effect
ED: Euclidean distance
KEGG: Kyoto encyclopedia of genes and genomes
GO: Gene ontology
FDR: False discovery rate
APPL2: Adaptor protein, phosphotyrosine interacting with PH domain and leucine zipper 2
NLRP12: NLR family pyrin domain containing 12
BNIP3L: BCL2 interacting protein 3 like
*EPHB2*: EPH receptor B2
SAMD9L: Sterile alpha motif domain containing 9 like
MAPK: Mitogen-activated protein kinase
ROS: Reactive oxygen species
BWA: Burrows-wheeler aligner
